# Echocardiographic assessment of pulmonary artery occlusion pressure in ventilated patients: a transoesophageal study

**DOI:** 10.1186/cc6792

**Published:** 2008-02-19

**Authors:** Philippe Vignon, Ali AitHssain, Bruno François, Pierre-Marie Preux, Nicolas Pichon, Marc Clavel, Jean-Pierre Frat, Hervé Gastinne

**Affiliations:** 1 Medical-surgical Intensive Care Unit, Dupuytren Teaching Hospital, 2 Ave. Martin Luther King, 87000 Limoges, France; 2 Centre d'Investigation Clinique, Dupuytren Teaching Hospital, 2 Ave. Martin Luther King, 87000 Limoges, France; 3 University of Limoges, Department of Medicine, 2 Rue du Dr Marcland, 87000 Limoges, France; 4 Medical Intensive Care Unit, Teaching Hospital, Rue Montalembert, BP 69, 63000 Clermont-Ferrand, France; 5 Unit of Clinical Research and Biostatistics, Dupuytren Teaching Hospital, 2 Ave. Martin Luther King, 87000 Limoges, France; 6 Medical Intensive Care Unit, Jean Bernard Teaching Hospital, Rue de la Miletrie, 86000 Poitiers, France

## Abstract

**Background:**

Non-invasive evaluation of left ventricular filling pressure has been scarcely studied in critically ill patients. Accordingly, we prospectively assessed the ability of transoesophageal echocardiography (TEE) Doppler to predict an invasive pulmonary artery occlusion pressure (PAOP) ≤ 18 mmHg in ventilated patients.

**Methods:**

During two consecutive 3-year periods, TEE Doppler parameters were compared to right heart catheterisation derived PAOP used as reference in 88 ventilated patients, haemodynamically stable and in sinus rhythm (age: 63 ± 14 years; simplified acute physiologic score (SAPS) II: 45 ± 12). During the initial period (protocol A), threshold values of pulsed-wave Doppler parameters to predict an invasive PAOP ≤ 18 mmHg were determined in 56 patients. Derived Doppler values were prospectively tested during the subsequent period (protocol B) in 32 patients.

**Results:**

In protocol A, Doppler parameters had similar area under the receiver operating characteristic (ROC) curve. In protocol B, mitral E/A ≤ 1.4, pulmonary vein S/D > 0.65 and systolic fraction > 44% best predicted an invasive PAOP ≤ 18 mmHg. Lateral E/E' ≤ 8.0 or E/Vp ≤ 1.7 predicted a PAOP ≤ 18 mmHg with a sensitivity of 83% and 80%, and a specificity of 88% and 100%, respectively. Areas under ROC curves of lateral E/E' and E/Vp were similar (0.91 ± 0.07 vs 0.92 ± 0.07: p = 0.53), and not significantly different from those of pulsed-wave Doppler indices.

**Conclusion:**

TEE accurately predicts invasive PAOP ≤ 18 mmHg in ventilated patients. This further increases its diagnostic value in patients with suspected acute lung injury/acute respiratory distress syndrome.

## Introduction

Estimation of left ventricular (LV) filling pressure by physical examination is unreliable[1]. Accordingly, the clinical evaluation of LV filling pressure currently relies on the invasive measurement of pulmonary artery occlusion pressure (PAOP) – a widely accepted surrogate of left atrial pressure – during right heart catheterisation (RHC) [2]. PAOP is not a reliable predictor of fluid responsiveness [3] but is diagnostic in patients who present with an acute respiratory failure and associated bilateral radiographic infiltrates. Currently proposed haemodynamic criterion for the diagnosis of acute lung injury (ALI) and acute respiratory distress syndrome (ARDS) is a pulmonary artery wedge pressure ≤ 18 mmHg [4], usually measured during RHC. This invasive procedure may lead to relevant complications [5] and is limited by confounding factors for the measurement of PAOP in ventilated patients [6].

Transoesophageal echocardiography (TEE) is being increasingly used in the intensive care unit (ICU) settings for the evaluation of critically ill patients with a circulatory or a respiratory failure [7]. Numerous clinical studies performed in spontaneously breathing heart failure patients have uniformly shown that echocardiography Doppler may accurately predict invasive PAOP [8-13]. By contrast, only few Doppler studies have yet been conducted in ventilated ICU patients [14-19], and frequently comprised patients with known cardiac disease [18,19]. Moreover, initial studies frequently attempted to estimate the absolute value of invasive PAOP using several Doppler indices combined in complex equations [8,9,13,18,19], whereas semi-quantitative evaluation of LV filling pressure based on simple yet robust, easy-to-measure parameters appears more adapted in the ICU environment. Finally, only two studies have previously assessed the ability of mitral and pulmonary venous flow Doppler to predict a PAOP ≤ 18 mmHg in ventilated ICU patients [14,15] and studies using DTI and colour Doppler indices have unfortunately focused on the prediction of lower levels of PAOP [16,17].

Accordingly, we prospectively evaluated the ability of TEE Doppler to accurately predict an invasive PAOP ≤ 18 mmHg in ventilated ICU patients.

## Materials and methods

Patients were enrolled in the study if they were mechanically ventilated, had a RHC already inserted and required a TEE examination, according to the current recommendations [20,21] and standard of care in our ICU [22]. Patients with non-sinus rhythm, atrioventricular conduction abnormalities, relevant mitral valvulopathy (that is, mitral stenosis, > grade II mitral regurgitation, mitral valve prosthesis) or a contra-indication to oesophageal intubation were excluded from this study.

This prospective study comprised two distinct protocols conducted during consecutive 3-year periods: protocol A evaluated the ability of TEE pulsed-wave Doppler parameters to predict a PAOP ≤ 18 mmHg in the first group of patients; protocol B prospectively tested conventional Doppler indices derived from protocol A in a second group of consecutive patients and assessed the potential additional diagnostic value of recently proposed Doppler tissue imaging (DTI) and colour Doppler indices to predict an invasive PAOP ≤ 18 mmHg. The protocols were approved by the Ethics Committee of the Société de Réanimation de Langue Française which waived the need of signed informed consent.

### Protocol A

A total of 56 ventilated ICU patients with stable haemodynamics (36 men; mean age (± SD): 66 ± 12 years; mean Simplified Acute Physiologic Score (SAPS) II: 47 ± 12) were enrolled into this protocol. Main indications for RHC were: circulatory failure in 29 patients (cardiogenic shock: *n *= 11; haemorrhagic or hypovolemic shock: *n *= 11; septic shock: *n *= 5; and other shock: *n *= 2) and acute respiratory failure in the 27 remaining patients (cardiogenic pulmonary oedema: *n *= 9; ALI: *n *= 5; ARDS: *n *= 8; and miscellaneous: *n *= 5). A SONOS 2500 system connected to a 5-Mhz multiplane TEE probe (Philips Ultrasound, Andover, MA, USA) with only pulsed-wave Doppler imaging capability was used in this protocol.

### Protocol B

A total of 32 haemodynamically stable patients (23 men; mean age: 57 ± 15 years; mean SAPS II: 41 ± 12) under ventilator conditions participated in this protocol. RHC was inserted for the assessment of an acute respiratory failure in 27 patients (cardiogenic oedema: *n *= 8; ALI: *n *= 6; ARDS: *n *= 10; interstitial pneumonitis: *n *= 2; and intra-alveolar haemorrhage: *n *= 1) and for shock in the remaining five patients. A SONOS 5500 upper-end platform connected to a 7-Mhz multiplane TEE probe (Philips Ultrasound, Andover, MA, USA) with DTI and colour M-mode capabilities was used in this protocol.

### TEE studies

All procedures were performed as previously described [22]. During the TEE study, neither blood volume expansion nor changes in catecholamine infusion rate was performed. In protocols A and B, the following parameters were recorded: LV fractional area change (FAC); maximal velocities and velocity time integrals of mitral Doppler E and A waves, and E wave deceleration time; maximal velocities and velocity time integrals of pulmonary vein Doppler S and D waves. E/A and S/D ratios were calculated and both the atrial filling fraction [23] and pulmonary vein systolic fraction [24] were computed.

In protocol B, a transoesophageal four-chamber view was used to place a 5-mm DTI sample volume at the lateral aspect of the mitral annulus and maximal early diastolic velocity (E' wave) was recorded in spectral pulsed mode. Colour M-mode recordings were obtained as previously described [25]. E/E' and E/Vp ratios were calculated.

All TEE measurements were independently performed off-line by the same trained operator (PV) with a level III in echocardiography [26], who was unaware of the value of PAOP measured with RHC. For each Doppler parameter, at least three end-expiratory measurements were performed on non-consecutive heartbeats, and averaged. Cardiac cycles with nonlinear deceleration slopes and fusion of early and late mitral flow velocity were excluded from the analysis. In our ICU, the inter-observer variability in the measurement of pulsed-wave Doppler indices ranges between 1 and 13%, and the intra-observer variability ranges between 2% and 7%, respectively [27]. Corresponding variability in the measurement of E' maximal velocity and Vp ranges between 4% and 11%, and between 2% and 7%, respectively [27].

### Invasive PAOP measurement

Adequate position of RHC in West's zone III was validated as previously described [28]. In order to perform RHC and TEE measurements in the same clinical settings, PAOP measurements and Doppler recordings were obtained within a 15-min time frame, and the ventilator was purposely not disconnected. Invasive PAOP was independently measured at end-expiration by an experienced investigator (AAH, BF, MC, NP, JPF) who was blinded to TEE results.

### Statistics

Patients' characteristics were compared between the two protocols using the Mann-Whitney test for continuous variables and the Fisher's exact test for qualitative variables. For the purpose of the current study, invasive PAOP measured using RHC was used as reference. Patients were divided into two groups according to the level of invasive PAOP ≤ or > 18 mmHg, and TEE Doppler parameters were compared between groups with the Mann-Whitney test in the two protocols.

In protocol A, receiver operating characteristic (ROC) curves were built in varying the discriminating threshold value of each pulsed-wave Doppler parameter to predict an invasive PAOP ≤ 18 mmHg, and the area under the curve (AUC) with its 95% confidence intervals (CI) were calculated. AUC were then compared between Doppler parameters using a Hanley-McNeil test [29]. To evaluate the potential influence of LV systolic function on the relationship between pulsed-wave Doppler parameters and invasive PAOP, patients were divided into two subsets based on LV FAC at the time of TEE examination: depressed LV systolic function (FAC ≤ 25%; *n *= 7) and preserved LV systolic function (FAC > 25%; *n *= 49). In the two subsets of patients, each Doppler value was compared to the corresponding value of invasive PAOP using Spearman's method, and results were then confronted. To take into account the known influence of age and heart rate on left cardiac diastolic Doppler velocity profiles, we assessed the potential influence of these parameters on the relationship between Doppler indices and invasive PAOP values using a logistic regression analysis.

Cut-off values of pulsed-wave Doppler parameters derived from protocol A were subsequently tested in another group of ventilated ICU patients studied in protocol B. Diagnostic accuracy of each pulsed-wave Doppler parameter for predicting an invasive PAOP ≤ 18 mmHg was determined conventionally. In addition, ROC curves were generated for newly proposed Doppler indices (E/E' and E/Vp), and areas under curves were compared to those of conventional Doppler parameters obtained in protocol B [29].

All results are presented as mean ± standard deviation and a p value of less than 0.05 was considered statistically significant.

## Results

### Study population

Among the 108 patients evaluated by both RHC and TEE during the study period, 20 were excluded because of non-sinus rhythm (*n *= 16) or relevant valvulopathy (*n *= 4). The study population comprised 88 ventilated patients (59 men; mean age: 63 ± 14 years; mean SAPS II: 45 ± 12) who were admitted to the ICU for a medical condition (*n *= 63) or a non-scheduled surgery (*n *= 25). Among them, 63 patients (72%) were receiving vasopressor therapy or inotropic support at the time of haemodynamic evaluation. ICU mortality was 26%. A total of 70 patients (80%) had an invasive PAOP ≤ 18 mmHg whereas the remaining 18 patients (20%) had a PAOP measured by RHC > 18 mmHg, the absolute value of invasive PAOP ranging between 3 and 27 mmHg. Haemodynamic and respiratory parameters recorded at the time of TEE examination in the study population are summarised in Table 1. In protocol A, patients were older, had higher severity score and lower blood pressure, and more frequently required vasopressor therapy than in protocol B. Patients in protocol B had higher pulmonary artery pressure and pulmonary vascular resistance when compared to their counterparts studied in protocol A (Table 1).

**Table 1:** Patients' characteristics and haemodynamic findings obtained by right heart catheterisation at the time of TEE study

	Protocol A (*n *= 56)	Protocol B (*n *= 32)
Age (years)	66 ± 12	57 ± 15^a^
Men (n)	36 (64)	23 (72)
SAPS II	47 ± 12	41 ± 12^a^
Reason for admission (n):		
Medical condition	37 (66)	26 (81)
Non scheduled surgery	19 (34)	6 (19)
Vasopressor or inotropic support (n)	47 (84)	16 (50)^a^
Blood pressure (mmHg):		
Systolic	116 ± 25	133 ± 22^a^
Diastolic	61 ± 16	72 ± 15^a^
Mean	79 ± 17	94 ± 15^a^
Heart rate (bpm)	105 ± 19	98 ± 22
RAP (mmHg)	11 ± 6	10 ± 4
PAP (mmHg):		
Systolic	35 ± 13	43 ± 10^a^
Diastolic	21 ± 6	22 ± 5
Mean	27 ± 8	30 ± 7^a^
PAOP (mmHg)	12 ± 5	14 ± 6
Cardiac index (L/min/m^2^)	3.0 ± 1.0	3.2 ± 1.4
PVR (dynes·s·cm^-5^)	424 ± 190	466 ± 201
SVR (dynes·s·cm^-5^)	1 948 ± 824	2 402 ± 1087
V_T _(mL/kg)	8 ± 2	8 ± 2
PEEP (cm H_2_O)	9 ± 4	10 ± 3

^a^p < 0.05. Numbers between brackets are percentages. Abbreviations: PAP, pulmonary artery pressure; PAOP, pulmonary artery occlusion pressure; PVR, pulmonary vascular resistance; RAP, right atrial pressure; SAPS: simplified acute physiologic score; SVR, systemic vascular resistance; TEE, transoesophageal echocardiography.

### Protocol A

**Figure 1:**
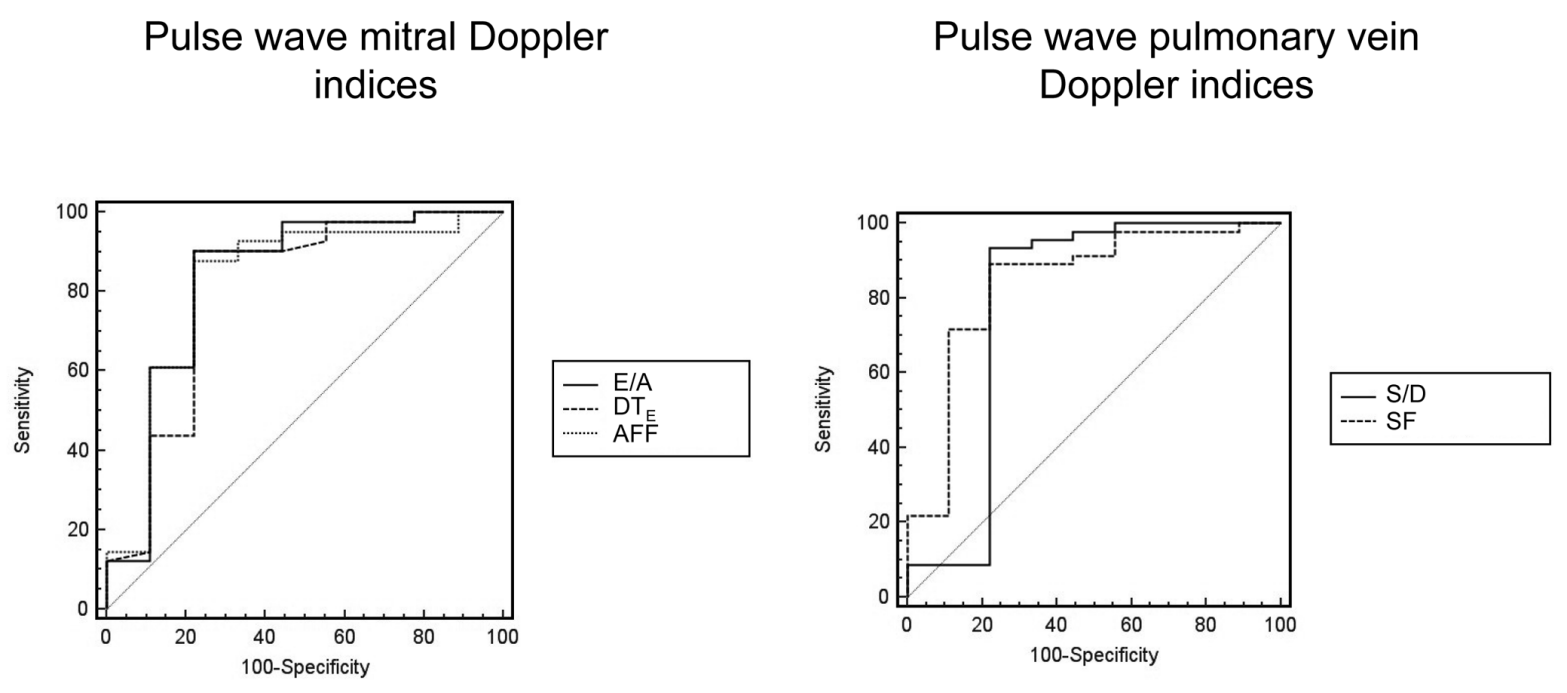
Receiver operating characteristic (ROC) curves of transoesophageal echocardiography (TEE) Doppler parameters to predict an invasive pulmonary artery occlusion pressure (PAOP) ≤ 18 mmHg in protocol A. Left panel: area under the curves (AUC) with standard error and 95% confidence intervals (CI) and p values (comparison of the actual AUC with the 0.50 AUC) of mitral Doppler parameters were as follows: E/A, 0.83 ± 0.09 (95% CI: 0.70–0.92; p = 0.0002); mitral E wave deceleration time, 0.81 ± 0.07 (95% CI: 0.67–0.90; p < 0.0001); atrial filling fraction: 0.82 ± 0.07 (95% CI: 0.68–0.91; p < 0.0001). Right panel: AUC with standard error and 95% CI of pulmonary vein Doppler parameters were the followings: S/D, 0.78 ± 0.07 (95% CI: 0.65–0.88; p = 0.0001); systolic fraction, 0.84 ± 0.06 (95% CI: 0.72–0.93; p < 0.0001). Abbreviations: AFF, atrial filling fraction; DT_E_, deceleration time of early diastolic mitral E wave; SF, systolic fraction.

Mean LV FAC was significantly lower in patients with elevated PAOP when compared to the subset of patients with LV filling pressure ≤ 18 mmHg (32 ± 14% vs 48 ± 15%: p = 0.001). Mitral Doppler parameters were not possible to measure in six patients secondary to fused E/A velocity profiles related to marked tachycardia, whereas pulmonary vein Doppler indices were obtained in all but one patient due to inadequate imaging quality. Patients with PAOP ≤ 18 mmHg had lower E/A ratios, but higher atrial filling fractions and prolonged E wave deceleration time when compared to patients with elevated LV filling pressure (Table 2). Both the S/D ratio and systolic fraction were higher in the presence of low PAOP (Table 2). Threshold values for predicting an invasive PAOP ≤ 18 mmHg were: E/A ≤ 1.4, DT_E _> 100 ms, atrial filling fraction > 31%, S/D > 0.65, and systolic fraction > 44%. Doppler parameters had similar areas under the ROC curves (Figure 1). Correlations between Doppler and PAOP values were consistently closer in the subset of patients with depressed LV systolic function, when compared to patients with preserved cardiac performance (Table 3). Using a logistic regression analysis, age and heart rate did not significantly alter the relationship between TEE Doppler indices and invasive PAOP (age: p = 0.62, odds ratio: 1.02, 95% CI: 0.94–1.12; heart rate: p = 0.76, odds ratio: 1.03, 95% CI: 0.86–1.23).

**Table 2:** Pulsed-wave Doppler findings obtained in protocol A, according to the level of invasive PAOP measured using RHC

	PAOP ≤ 18 mmHg (*n *= 46)	PAOP > 18 mmHg (*n *= 10)	p Value
Mitral Doppler:			
E/A	0.9 ± 0.4	2.0 ± 1.0	0.002
DT_E _(ms)	179 ± 72	117 ± 68	0.005
Atrial filling fraction (%)^a^	47 ± 15	31 ± 14	0.003
Pulmonary vein Doppler:			
S/D	1.3 ± 0.5	0.7 ± 0.7	0.008
Systolic fraction (%)^b^	59 ± 14	38 ± 17	0.001

^a^VTI A/(VTI A + VTI E) ·100; ^b^VTI S/(VTI S + VTI D) ·100. Abbreviations: DT_E_, deceleration time of early diastolic mitral E wave; PAOP, pulmonary artery occlusion pressure; RHC, right heart catheterisation; VTI, velocity time integral.

**Table 3:** Linear correlation between the values of pulsed-wave Doppler parameters and invasive PAOP according to LV systolic function in patients enrolled in protocol A

	LV systolic dysfunction (*n *= 7)		Preserved LV systolic function (*n *= 49)	
Pulsed-wave Doppler indices	Spearman rho coefficient	p Value	Spearman rho coefficient	p Value

E/A	1.00	0.025	0.26	0.090
DT_E_	-0.94	0.035	-0.08	0.580
AFF	-0.94	0.035	-0.25	0.090
S/D	-0.96	0.018	-0.22	0.130
SF	-0.86	0.036	-0.31	0.034

Abbreviations: AFF, atrial filling fraction; DT_E_, deceleration time of early diastolic mitral E wave; LV, left ventricle; PAOP, pulmonary artery occlusion pressure; SF, systolic fraction.

### Protocol B

**Figure 2:**
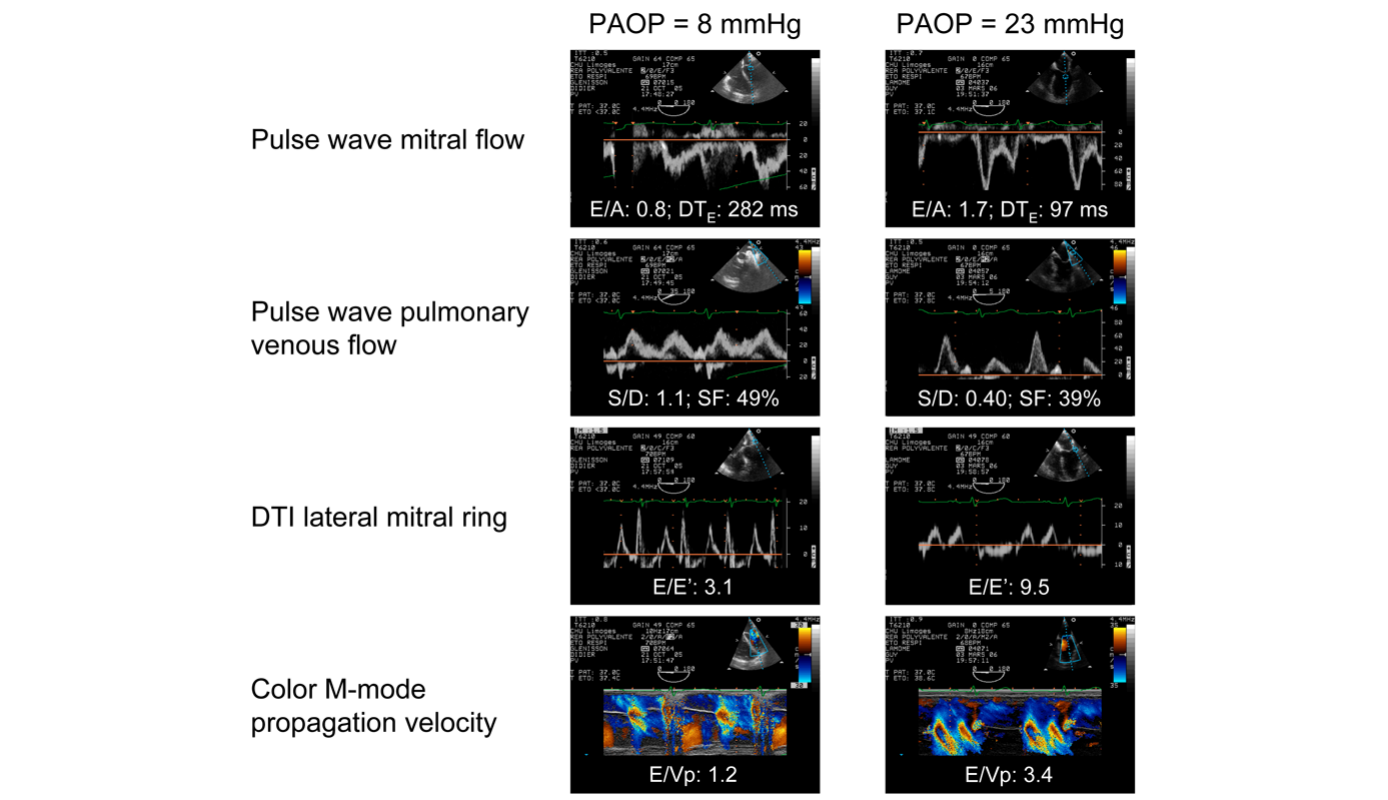
Examples of transoesophageal echocardiography (TEE) Doppler findings obtained in a patient with a low invasive pulmonary artery occlusion pressure (PAOP) (left panels) and in a patient with elevated left ventricle (LV) filling pressures (right panels) who were both enrolled in protocol B. Values of studied Doppler indices are noted on each corresponding TEE view. Abbreviations: DTI, Doppler tissue imaging; DT_E_, deceleration time of early diastolic mitral E wave; SF, systolic fraction.

Patients with elevated PAOP also exhibited significantly lower LV FAC than patients with PAOP ≤ 18 mmHg (29 ± 8% vs 44 ± 16%: p = 0.02). Illustrative examples of TEE Doppler findings obtained in the two subsets of patients according to the level of invasive PAOP are shown in Figure 2. Among the threshold values of Doppler parameters initially determined in protocol A, a mitral E/A ratio ≤ 1.4, a pulmonary vein S/D ratio > 0.65 and a systolic fraction > 44% allowed us to best subsequently predict an invasive PAOP ≤ 18 mmHg in protocol B (Table 4). Vp was not possible to obtain in five patients whereas E' maximal velocity of lateral mitral annulus was adequately recorded in all cases. Mean Vp and E' maximal velocity recorded at the lateral mitral ring were significantly higher in patients with PAOP ≤ 18 mmHg, when compared to those with elevated LV filling pressure (Table 5). By contrast, mean lateral E/E' and E/Vp ratios were significantly lower in the presence of an invasive PAOP ≤ 18 mmHg (Table 5). A lateral E/E' ratio ≤ 8.0 allowed predicting an invasive PAOP ≤ 18 mmHg with a sensitivity of 83% (95% CI: 63–95%) and a specificity of 88% (95% CI: 47–98%), while an E/Vp ratio ≤ 1.7 had a 80% sensitivity (95% CI: 56–94%) and a 100% specificity (95% CI: 59–100%) for the prediction of low LV filling pressures. Area under the ROC curves of lateral E/E' and E/Vp ratios were similar (0.91 ± 0.07, 95% CI: 0.76–0.98 vs 0.92 ± 0.07, 95% CI: 0.75–0.99; p = 0.53), and not significantly different when compared to those of pulsed-wave Doppler indices (data not shown).

**Table 4:** Diagnostic accuracy of cut-off values of pulsed-wave Doppler indices derived from protocol A and prospectively tested in protocol B for predicting an invasive PAOP ≤ 18 mmHg

Pulsed-wave Doppler indices derived from protocol A	Sensitivity (%)	Specificity (%)	Positive predictive value (%)	Negative predictive value (%)
E/A = 1.4	75 (53–89)	100 (60–100)	100 (78–100)	57 (30–81)
DT_E _> 100 ms	81 (57–94)	63 (26–90)	85 (61–96)	56 (23–85)
AFF^a ^> 31%	79 (57–92)	63 (26–90)	86 (64–96)	50 (20–80)
S/D > 0.65	96 (77–100)	100 (60–100)	100 (82–100)	89 (51–99)
SF^b ^> 44%	92 (71–99)	88 (47–99)	96 (76–100)	78 (40–96)

^a^VTI A/(VTI A + VTI E) ·100; ^b^VTI S/(VTI S + VTI D) ·100. Numbers between brackets indicate 95% confidence intervals. Abbreviations: AFF, atrial filling fraction; DT_E_, deceleration time of early diastolic mitral E wave; PAOP, pulmonary artery occlusion pressure; SF, systolic fraction; VTI, velocity time integral.

**Table 5:** Doppler indices based on DTI of the lateral mitral ring and colour M-mode propagation velocity obtained in protocol B, according to the level of invasive PAOP measured during RHC

	PAOP ≤ 18 mmHg (*n *= 24)	PAOP > 18 mmHg (*n *= 8)	p Value
Vp (cm/s)	45 ± 10	35 ± 8	0.05
E' lateral (cm/s)	12.9 ± 3.9	9.2 ± 1.3	0.01
E/Vp	1.7 ± 0.6	2.7 ± 0.5	0.0006
E/E' lateral	5.9 ± 2.2	10.6 ± 3.3	0.0002

Abbreviations: DTI, Doppler tissue imaging; PAOP, pulmonary artery occlusion pressure; RHC, right heart catheterisation; Vp, propagation velocity of early diastolic blood flow within the LV measured using colour M-mode.

## Discussion

Current haemodynamic criterion for the diagnosis of ALI/ARDS relies on a pulmonary artery wedge pressure ≤ 18 mmHg [4]. RHC is traditionally used to evaluate patients with circulatory or respiratory failure, as reflected by the heterogeneity of our study population. During the past decade, TEE progressively supplanted RHC for the assessment of circulatory failure in numerous ICUs [7,30-33]. Nevertheless, RHC provides direct measurement of PAOP, which is not accessible when using newer invasive monitoring systems. Although less used, RHC remains of value for assessing patients presenting with acute respiratory failure (protocol B). In these ventilated ICU patients, the current study showed that simple yet robust TEE Doppler indices allow predicting accurately a level of invasive PAOP ≤ 18 mmHg. The additional advantage of TEE is its ability to comprehensively depict heart-lung interactions under mechanical ventilation [34]. However, TEE evaluation of LV filling pressure may be limited by tachycardia or the presence of non-sinus rhythm which both preclude obtaining certain Doppler parameters, as in approximately 20% of our screened patients.

Mitral E velocity is primarily determined by early diastolic trans-mitral pressure gradient while A velocity reflects the atrial contribution to late diastolic LV filling. In the present study, a mitral E/A ratio ≤ 1.4 was specific and had a 75% sensitivity to predict an invasive PAOP ≤ 18 mmHg. A higher threshold value (≥ 2) was proposed to detect elevated PAOP (> 18 or ≥ 20 mmHg) in previous studies conducted in ICU patients [15] and in cardiac patients [10], with also a high positive predictive value and specificity but a lower sensitivity. In our patients, this simple Doppler parameter was more accurate than both the atrial filling fraction [23] and mitral E wave deceleration time [10] to semi-quantitatively evaluate invasive PAOP.

The pulmonary venous flow Doppler pattern mirrors the changes of left atrial pressure and has long been proposed to estimate LV filling pressure in ventilated patients [24]. In ARDS patients, Vargas *et al*. [14] have shown that a TEE systolic fractio *n *= 40% allowed predicting an invasive PAOP > 18 mmHg with a positive predictive value of 100% (95% CI: 52–100%). Similarly, we found that a systolic fraction > 44% accurately predicts an RHC-derived PAOP ≤ 18 mmHg with a 96% positive predictive value (95% CI: 76–100%). In ventilated ICU patients, Boussuges *et al*. [15] reported a 55% positive predictive value of a systolic fraction < 40% to identify patients with a PAOP > 18 mmHg using transthoracic echocardiography. These discrepant results are presumably explained by the superiority of TEE for studying pulmonary venous flow [12,19], as reflected by the exclusion of 29% of eligible patients from data analysis in the study by Boussuges *et al*. due to poor imaging quality [15].

As previously reported in cardiac patients [8,9,35,36], the relationship between Doppler indices and invasive PAOP was closer in our patients with LV systolic dysfunction. This would help the physician to confidently identify a cardiogenic pulmonary oedema in the presence of Doppler velocity profiles consistent with elevated PAOP since heart failure patients typically exhibit high LV filling pressures.

DTI early diastolic velocity of the lateral mitral ring and colour M-mode propagation velocity are linked to the rate of LV relaxation but appear relatively preload-independent [25,37,38]. Accordingly, their combination with mitral E wave velocity has been proposed for the evaluation of LV filling pressure [36,38]. In the present study, both E/E' and E/Vp ratios failed to better identify ventilated ICU patients with invasive PAOP ≤ 18 mmHg than single pulsed-wave Doppler parameters. This may be potentially explained by a relatively low prevalence of severe LV diastolic dysfunction in our study population since only six patients who participated in protocol B (19%) had a maximal E' velocity < 8 cm/s [39], and mean Vp in the subset of 17 patients (53%) with decreased propagation velocity (< 45 cm/s [39]) reached 37 ± 6 cm/s (range: 21–44 cm/s). Accordingly, the influence of impaired LV relaxation has presumably minimally altered mitral E wave maximal velocity, thus offsetting the potential additional value of combining this conventional Doppler parameter to a preload-independent Doppler index that better reflects LV diastolic properties [38,40,41]. This hypothesis has already been raised in a study of healthy volunteers where the relationship between mitral E wave velocity and PAOP was the strongest of all measured Doppler variables, including combined indices E/E' and E/Vp [41]. Accordingly, combined Doppler indices appear to be of additional value for estimating LV filling pressure in patients with underlying cardiac diseases known to alter diastolic properties.

The current study has several limitations. Although particular attention was directed towards the precise measurement of PAOP during RHC, this gold standard suffers from intrinsic substantial well-identified limitations [6] that could have altered the relationship between Doppler indices and invasive PAOP values. Heart rate and age, which are known to physiologically influence Doppler flow patterns [42], have not been taken into account in our data analysis. Nevertheless, using a logistic regression analysis, these variables were not identified as confounding factors for TEE Doppler prediction of invasive PAOP in patients studied in protocol A. Although conventional Doppler parameters were derived from an inhomogeneous group of patients (protocol A), they were prospectively tested in a subset of patients with respiratory failure (protocol B). The proposed threshold values of E/E' and E/Vp could not be prospectively tested since DTI and high-quality colour M-mode were not available in protocol A. Lateral E' velocity was only studied since we [27] and others [43,44] have shown that E' velocity was preload sensitive when recorded at the septal portion of the mitral ring. Finally, a single determination of PAOP was performed since we attempted no intervention to induce variations in LV loading conditions. Nevertheless, previous studies have already shown in ventilated ICU patients that Doppler-derived indices accurately track treatment-induced variations of PAOP [16,17].

## Conclusion

In the present study, simple TEE Doppler parameters identified patients presenting with an acute respiratory failure associated with an invasive PAOP ≤ 18 mmHg. In our study population, the additional use of DTI early diastolic velocity of lateral mitral ring and colour M-mode Doppler propagation velocity failed to increase TEE diagnostic accuracy. Accurate evaluation of LV filling pressures further increases the diagnostic value of TEE for routine assessment of ventilated patients with suspected ALI/ARDS.

## Key messages

Simple pulsed-wave Doppler indices (mitral E/A ≤ 1.4, pulmonary vein S/D > 0.65 and systolic fraction > 44%) allow the prediction of an invasive PAOP ≤ 18 mmHg in ventilated patients.

Lateral E/E' ≤ 8.0 or E/Vp ≤ 1.7 predicted a PAOP ≤ 18 mmHg with a sensitivity of 83% and 80%, and a specificity of 88% and 100%, respectively.

Diagnostic accuracy of combined indices such as lateral E/E' and E/Vp was similar (areas under ROC curves 0.91 ± 0.07 vs 0.92 ± 0.07: p = 0.53), and not significantly different from those of pulsed-wave Doppler parameters.

## Abbreviations

ARDS = acute respiratory distress syndrome; ALI = acute lung injury; PAOP = pulmonary artery occlusion pressure; TEE = transoesophageal echocardiography.

## Competing interests

The authors declare that they have no competing interests.

## Authors' contributions

PV, PMP and HG designed the study. AAH, BF, NP and MPF contributed to patient enrolment and invasive haemodynamic measurements. All authors contributed to the preparation of the manuscript.
